# Lactylation of tau in human Alzheimer's disease brains

**DOI:** 10.1002/alz.14481

**Published:** 2024-12-30

**Authors:** Xiaoyu Zhang, Yan Liu, Michaella J. Rekowski, Ning Wang

**Affiliations:** ^1^ Department of Cell Biology and Physiology University of Kansas Medical Center Kansas City Kansas USA; ^2^ Institute of Reproductive and Developmental Sciences University of Kansas Medical Center Kansas City Kansas USA; ^3^ Mass Spectrometry/Proteomics Core Laboratory University of Kansas Medical Center Kansas City Kansas USA; ^4^ Department of Cancer Biology University of Kansas Medical Center Kansas City Kansas USA; ^5^ Landon Center on Aging University of Kansas Medical Center Kansas City Kansas USA; ^6^ University of Kansas Alzheimer's Disease Research Center Fairway Kansas USA

**Keywords:** Alzheimer's disease, lactate, lactylation, posttranslational modification, tau, tauopathy

## Abstract

**INTRODUCTION:**

Aggregation of hyperphosphorylated tau (tauopathy) is associated with cognitive impairment in patients with Alzheimer's disease (AD). In AD, a metabolic shift due to the Warburg effect results in increased lactate production. Lactate can induce a post‐translational modification (PTM) on proteins that conjugates lactyl groups to lysine (K) residues, which is known as lactylation.

**METHODS:**

We analyzed lactylation of tau in control and AD brain tissue and conducted cell‐based assays. In addition, we used in vitro assays to determine whether p300 catalyzed tau lactylation.

**RESULTS:**

Quantitative proteomics detected that tau lactylation was elevated in AD brains, with K residue at position 331 (K331) being a prominent site. Lactate induced tau lactylation, which increased tau phosphorylation and cleavage and reduced ubiquitination. Inhibition of lactate production lowered tau lactylation; p300 catalyzed tau lactylation.

**DISCUSSION:**

Our findings suggest that tau lactylation links metabolic dysregulation with tauopathy and could serve as a novel diagnostic and therapeutic target.

**Highlights:**

- Elevated tau lactylation, particularly at K331, is evident in in human AD brain samples.
- Lactate induces tau lactylation, enhancing phosphorylation and cleavage while inhibiting ubiquitination.
- The acetyl‐transferase p300 catalyzes tau lactylation, with K331 being the most prominent site.

## 1 BACKGROUND

A key feature in Alzheimer's disease (AD) pathogenesis is metabolic dysfunction.^1^ Originally observed in cancer cells, the Warburg effect,^2^ has received increasing attention. This effect entails a preferential shift toward aerobic glycolysis over oxidative phosphorylation, even in the presence of oxygen. As such, most glucose is converted to lactate, which is then supplied to neurons as an alternative fuel in AD.^3^, ^4^

In addition to its metabolic function, lactate has been shown to induce a novel post‐translational modification (PTM) on histones and proteins known as “lactylation,” which is the addition of a lactyl group to lysine (K) residues.^5^ Lactylation of lysine residues in histones functions as an epigenetic modification to regulate gene transcription.^5^ In the context of AD, it has been reported that elevated histone H4 lysine 12 lactylation (H4K12la) levels in microglia near amyloid beta (Aβ) plaques enhance glycolytic gene transcription, which worsens microglial dysfunction in AD.^6^ More recently, protein lactylation on lysine residues has been detected, and protein lactylation as a PTM has been shown to play crucial roles in regulating their biological functions.^7^ However, whether protein lactylation is implicated in AD remains unclear.^8^

Amyloid plaques and neurofibrillary tangles (NFTs) are neuropathological hallmarks of AD. Although amyloid plaques are composed of Aβ peptides, NFTs are composed of intracellular aggregates of hyperphosphorylated microtubule‐binding protein tau (tauopathy). Abnormal accumulation of hyperphosphorylated tau as insoluble NFTs is implicated in the pathogenesis of AD as well as other neurodegenerative disorders involving tauopathy. Of the two pathological features, amyloid plaques and NFTs, the presence of NFTs strongly correlates with symptoms such as cognitive decline in patients with AD. Notably, tau is profoundly regulated by PTMs, for example, phosphorylation, ubiquitination, and acetylation, and is profoundly implicated in tauopathy.^9^

Here, we have identified lactylation as a novel PTM of tau. Elevated tau lactylation was observed in human AD brain samples, with K residue at position 331 (K331) being a prominent site that is associated with AD pathogenesis. Functionally, our data suggest that tau lactylation regulates its phosphorylation, ubiquitination, turnover, and cleavage. The findings suggest that increased lactate levels in AD brains may drive tau lactylation, potentially contributing to tauopathy and offering a new diagnostic and therapeutic target.

## 2 METHODS

### 2.1 Patients’ samples

Autopsy brain tissue samples from the frontal cortex were provided by the Neuropathology Core of the University of Kansas Alzheimer's Disease Research Center (KU ADRC). These samples were homogenized on dry ice and subsequently aliquoted for analysis. Detailed information regarding the human samples utilized in this study, including the AD and control cases, is provided in Table S1.

**RESEARCH IN CONTEXT**

1. **Systematic review**: Hyperphosphorylation of tau is a well‐established pathological hallmark of Alzheimer's disease (AD). Tau undergoes various post‐translational modifications (PTMs) that influence its function, including lactylation, a PTM induced by lactate. Although metabolic shifts such as increased lactate production are known to occur in AD, a review of the literature via PubMed reveals that tau lactylation has not been explored previously in the context of AD.
2. **Interpretation**: Our study demonstrates that tau lactylation, particularly at K331, is elevated in AD brains. Lactate induces tau lactylation, promoting tau phosphorylation and cleavage while reducing ubiquitination. We identified p300 as the enzyme responsible for catalyzing this modification.
3. **Future directions**: This discovery links metabolic dysregulation in AD, specifically increased lactate production, with tauopathies, suggesting a novel mechanism and therapeutic target. Future research using genetic AD mouse models and AD patient–derived neurons will be crucial for assessing the physiological and pathological significance of tau lactylation and evaluating the therapeutic potential of targeting lactate metabolism or tau modifications.

### 2.2 Antibodies

Anti‐L‐lactyllysine (PTM‐1401RM) antibody was purchased from PTMBIO. Anti‐AT8 (MN1020) antibody was purchased from Thermo Fisher Scientific. Anti‐tau (210‐241) (Tau‐5, MAB361), anti‐tau^368N^ (ABN‐1703), anti‐Flag tag (F1804), and anti‐HA tag (H6908) antibodies were purchased from Sigma. Anti‐GFP (ab290) antibody was purchased from Abcam. Anti‐Actin (66009‐1‐Ig), Anti‐His tag (66005‐1‐Ig), and Anti‐pan‐acetylation (66289‐1‐Ig) antibodies were purchased from Proteintech Group. Information on these antibodies, including their dilutions used in the experiments, is provided in Table S2.

### 2.3 Plasmids

Tau^WT^ plasmid was kindly provided by the Laura J. Blair lab. pLKO.3G was a gift from Christophe Benoist & Diane Mathis (Addgene plasmid #14748). pRK5‐EGFP‐Tau AP was a gift from Karen Ashe (Addgene plasmid #46905).^10^ pSG5‐HA‐p300 was a gift from Elizabeth Wilson (Addgene plasmid #89094).^11^ HA‐Ubiquitin was a gift from Edward Yeh (Addgene plasmid #18712).^12^ pAdDeltaF6 was a gift from James M. Wilson (Addgene plasmid #112867). pAAV2/8 was a gift from James M. Wilson (Addgene plasmid #112864). AAV.CBA.eGFP.2A.wtTau was a gift from Bradley Hyman (Addgene plasmid #140424).^13^ Tau^3KR^ plasmid was generated at Gene Universal.

### 2.4 Cell line

Human embryonic kidney (HEK) 293T cell lines were procured from the American Type Culture Collection (ATCC). Cells were maintained in a humidified incubator set at 37°C with 5% CO_2_. Cells underwent validation via short tandem repeat (STR) DNA profiling and were confirmed to be free of mycoplasma contamination through polymerase chain reaction (PCR) testing. Culture media (Dulbecco's Modified Eagle Medium [DMEM]) were supplemented with 10% fetal bovine serum (FBS) and 1% penicillin‐streptomycin to support optimal growth conditions. Mouse primary neuronal cultures were prepared from cortices of postnatal day 1 pups. Cells were dissociated and plated on dishes coated with 0.1 mg/mL poly‐D‐lysine. Cells were cultured in neurobasal medium supplemented with B27. Experimental treatments were conducted at 7–13 days in vitro (DIV) in neurobasal medium supplemented with N2. Recombinant AAV (adeno‐associated virus)‐Tau^WT^ was generated by co‐transfection of the AAV plasmids with helper plasmids, including the AAV2/8 vector, pAdDeltaF6 vector, and AAV.CBA.eGFP.2A.wtTau vector, into 293T cells. AAV was collected at 72 h and was used to infect primary neurons. For western blot assays, cells were treated with various compounds under the following conditions: L‐lactate at 2 mM, sodium dichloroacetate (DCA) at 20 mM, 2‐deoxy‐d‐glucose (2‐DG) at 10 mM, rotenone at 10 mM, cycloheximide (CHX) at 100 mg/mL, MG132 at 20 µM. All treatments were applied for a duration of 24 h.

Transient transfection of DNA plasmids into HEK 293T cells was performed using Lipofectamine 3000 (Thermo Fisher Scientific). Six hours post‐transfection, the media was replaced with fresh complete media. For lentiviral transduction, HEK 293T were infected with control or *LDHA* shRNA lentivirus. Harvested lentiviruses were added to the cells for further experiments with 8 µg/mL polybrene to enhance infection efficiency.

### 2.5 Western blot analysis

Total protein was extracted using radioimmunoprecipitation assay (RIPA) buffer supplemented with 1 mM phenylmethylsulfonyl fluoride (PMSF) (Sigma) and a protease inhibitor cocktail (Sigma P8340). Following centrifugation at 12,000 × *g* for 15 min at 4°C, the lysates were collected. The pellet was washed with ice‐cold RIPA buffer, followed by lysis in 10% sodium dodecyl sulfate (SDS) buffer (10% SDS, 250 mM Tris, pH 6.8). The lysates were sonicated in a water bath sonicator until no particulate material remained. Protein concentrations were determined using the bicinchoninic acid (BCA) method for both RIPA‐soluble and RIPA‐insoluble fractions. Equal amounts of protein from each sample were mixed with lithium dodecyl sulfate (LDS) sample buffer (Invitrogen) and reducing agent (Invitrogen), then denatured at 70°C for 10 min. The samples were resolved on 4–12% Bis‐Tris gels (Thermo Fisher) and transferred to polyvinylidene fluoride (PVDF) membranes. Membranes were incubated overnight at 4°C with primary antibody at a 1:1,000 dilution, followed by washing and incubation with a secondary antibody. Protein detection was carried out using Clarity ECL Western Blotting Substrate (Bio‐Rad).

### 2.6 Immunohistochemistry and immunofluorescence

For immunohistochemistry, formalin‐fixed, paraffin‐embedded (FFPE) human tissue sections were dewaxed and rehydrated through a graded ethanol series, followed by microwave‐assisted antigen retrieval in 0.01 M citrate buffer (pH 6.0) and methanol/H₂O₂ treatment. Non‐specific binding was blocked with 5% goat serum before incubation with primary antibody and horseradish peroxidase (HRP)‐conjugated secondary antibodies. Staining was visualized using the NovaRed substrate kit (Vector, SK‐4800).

### 2.7 Immunoprecipitation and pull‐down

For transient transfection and co‐immunoprecipitation assays, HEK 293T cells were co‐transfected with plasmids encoding green fluorescent protein (GFP)‐tagged wild‐type tau or HA‐tagged p300. The transfected cells were lysed on ice for 25 min using NETN buffer (20 mM Tris‐HCl, pH 8.0, 100 mM NaCl, 1 mM ethylenediaminetetraacetic acid (EDTA), and 0.5% Nonidet P‐40) supplemented with 1 × protease inhibitors. Following centrifugation at 12,000 rpm for 10 min to remove cell debris, the soluble fractions were collected and incubated with hemagglutinin (HA) beads for 2 h at 4°C. The beads were washed three times with NP‐40‐EDTA‐Tris‐NaCl (NETN) buffer, then boiled in LDS loading buffer for 10 min and subjected to sodium dedecyl sulfate‐polyacrylamide gel electrophoresis (SDS‐PAGE). Membranes were blocked with 5% milk in tris buffered saline with Tween 20 (TBST) buffer and probed with indicated antibodies.

For in vitro pull‐down assay, His‐tagged purified tau protein and Flag‐tagged p300 were added to the NETN buffer. The mixture was incubated for 30 min at 4°C. Subsequently, Flag beads were added to capture the protein complexes and incubated for an additional 30 min at 4°C. The beads were washed three times with the NETN buffer and then denatured at 70°C for 10 min.

### 2.8 Flow cytometry

Single‐cell suspensions were prepared from cultured cells after incubation in serum‐free DMEM media containing 100 µM SCOTfluor lactic acid probe 510 for 30 min at 37°C. Then cells were washed in phosphate‐buffered saline (PBS) and stained with 4',6‐diamidino‐2‐phenylindole (DAPI) to exclude dead cells. Flow cytometry analysis was performed using a BD FACS Aria III.

### 2.9 In vitro tau lactylation and acetylation

The recombinant His‐tagged tau proteins (Abcam) were incubated with Flag‐tagged p300 proteins (R&D Systems) in reaction buffer (50 mM 4‐(2‐hydroxyethyl)‐1‐piperazineethanesulfonic acid [HEPES], pH7.8, 30 mM KCl, 0.25 mM EDTA, 5.0 mM MgCl2, 5.0 mM sodium butyrate, 2.5 mM dithiothreitol [DTT]) with 20 µM acetyl‐coenzyme A (CoA) (Sigma) or lactyl‐CoA (MCE). Reactions were incubated at 30°C for 30 min. Next, LDS loading buffer was added to the reaction and denatured at 70°C for 10 min. Samples were separated by SDS‐PAGE and immunoblotted with indicated antibodies.

### 2.10 In vitro tau ubiquitination

The recombinant tau proteins (Abcam) were ubiquitinated using the CHIP ubiquitin ligase kit (Misfolder Protein Ubiquitination Kit, BPBio Cat. #J5110) in the presence of E1, E2 (UBE2D3), and E3 CHIP enzymes. Briefly, 6 µL of recombinant tau protein (1 µg) was incubated with 3 µL reaction buffer, 1µL 10x E1 enzyme, 1µL 10x E2 (UBE2D3) enzyme, and 1µL 10x CHIP to initiate ubiquitination. The mixture was vortexed, briefly spun down, and incubated at 37°C for 6 h. To terminate the reaction, the tubes were placed on ice for 10 min, followed by heat inactivation at 70°C for 15 min.

### 2.11 In vitro tau cleavage

The recombinant tau proteins (Abcam) were incubated with rAEP proteins (Sino Biological) in reaction buffer. Reactions were incubated at 37°C for 30 min. Next, LDS loading buffer was added to the reaction and denatured at 70°C for 10 min. Samples were separated by SDS‐PAGE and immunoblotted with indicated antibodies.

### 2.12 Docking

The structure of lactyl‐CoA (CHEBI ID: 15529) was obtained in SMILES format from the ChEBI database (https://www.ebi.ac.uk/chebi/). This format was then converted to PDB format using Chem3D for subsequent molecular docking studies. Receptor proteins were downloaded in PDB format p300 (PDB: 6GYR). Protein preparation was performed using AutoDock Tool 4.2.1, and molecular docking was carried out with AutoDock Vina, which automatically calculates grid maps. Following successful docking, ligand binding positions and bond distances were analyzed and confirmed using PyMOL (www.pymol.org) and LigPlot. Both PyMOL and LigPlot enabled detailed visualization of ligand–protein interactions, including polar bonds, bond distances, and hydrophobic interactions.

### 2.13 Data analysis of mass spectrometry

Data were downloaded from ProteomeXchange (PXD020517) and searched with Proteome Discoverer 3.0^14^ using the Sequest algorithm against the human tau protein sequence and a database of common contaminants. Precursor mass tolerance was set to 10 ppm and fragment mass tolerance was set to 0.02 Da. The data were searched allowing a maximum of four modifications per peptide including variable modifications: oxidation (Met, +15.9949), acetylation (Lys, +42.0105), ubiquitination (Lys, +114.0429), lactylation (Lys, +72.0211), phosphorylation (Ser, Thr, Tyr, +79.9663), and static modification on cysteine (propionamide, +71.0371).

### 2.14 Mass spectrometry analysis of in vitro lactylated tau

After incubation of tau with p300, proteins were alkylated with iodoacetamide (10 mM final) in the dark for 10 min at room temperature. Ice‐cold acetone was added (1:4) to the sample and incubated overnight at −20°C to precipitate the proteins. The proteins were pelleted by centrifuging at 14,000 × *g* for 30 min at 4°C. The protein pellet was washed 2x with ice‐cold acetone and the pellet dried on the benchtop for 10 min. The pellet was resuspended in 50 mM tetraethylammonium bromide (TEAB), 2 mM CaCl_2_ buffer prior to digestion with trypsin overnight at 37°C with shaking at 500 RPM (Thermomixer, Eppendorf). The digestion was quenched with the addition of formic acid to 1%. Peptides were quantitated by Nanodrop spectrophotometry at 205 nm before liquid chromatography tandem mass spectrometry (LC‐MS/MS) analysis.

The peptide sample was injected using the Vanquish Neo (Thermo) nano‐UPLC onto a C18 trap column (PepMap Neo Trap, 0.3 mm × 5 mm, 5 µm particle size) using pressure loading. Peptides were eluted onto the separation column (PepMap Neo, 75 µm × 150 mm, 2 µm C18 particle size, Thermo) prior to elution directly to the mass spectrometer. Briefly, peptides were loaded and washed for 5 min at a flow rate of 0.350 µL/min at 2% B (mobile phase A: 0.1% formic acid in water, mobile phase B: 80% ACN, 0.1% formic acid in water). Peptides were eluted over 60 min from 2%–30% mobile phase B before ramping to 45% B in 5 min. The column was washed for 10 min at 100% B before re‐equilibrating at 2% B for the next injection. The nano‐LC was directly interfaced with the Orbitrap Ascend Tribrid mass spectrometer (Thermo) using a silica emitter (20 µm i.d., 10 cm, CoAnn Technologies) equipped with a high field asymmetric ion mobility spectrometry (FAIMS) source (Thermo). The data were collected by data‐dependent acquisition with the intact peptide detected in the Orbitrap at 120,000 resolving power from 375–1500 *m/z*. Peptides with charge +27 were selected for fragmentation by higher energy collision dissociation (HCD) at 28% NCE and were detected in the Orbitrap at 30,000 resolving power. Dynamic exclusion was set to 60 s after one instance. The mass list was shared between the FAIMS compensation voltages. FAIMS voltages were set at −45 (1.4 s), −60 (1 s), and −75 (0.6 s) CV for a total duty cycle time of 3 s. Source ionization was set at +1700 V with the ion transfer tube temperate set at 305°C. Raw files were searched against the human tau protein sequence and a common contaminants database using SEQUEST in Proteome Discoverer 3.0^14^. Abundances, abundance ratios, and *p*‐values were exported to Microsoft Excel for further analysis. Spectra were exported directly from Proteome Discoverer 3.0.

### 2.15 Quantification and statistical analysis

All experiments were replicated at least three times independently. Quantitative data from the experimental replicates were pooled and are presented as mean  ±  standard error (SE) as indicated in the figure legend. Normality testing was conducted using Kolmogorov–Smirnov tests. Compiled data were analyzed by Student's *t‐*test and two‐way analysis of variance (ANOVA).

## 3 RESULTS

### 3.1 Elevated lactylation of tau in the human AD brain

To identify AD‐associated changes in the expression of lactate signature genes (genes encoding regulators of lactate metabolism), we analyzed a single‐nuclei RNA‐seq (snRNA‐seq) data set from control and AD human brain samples.^15^ Pooled cell transcriptomes from ND and AD samples were used to define seven clusters (Figure S1A). It is important to note that cells from inhibitory (INH) neurons, excitatory (EX) neurons, oligodendrocytes (ODCs), oligodendrocyte progenitor cells (OPCs), and microglia (MG) clusters exhibit a significant and coordinated upregulation of lactate signature gene expression (Figure S1B and S1C).

**FIGURE 1:**
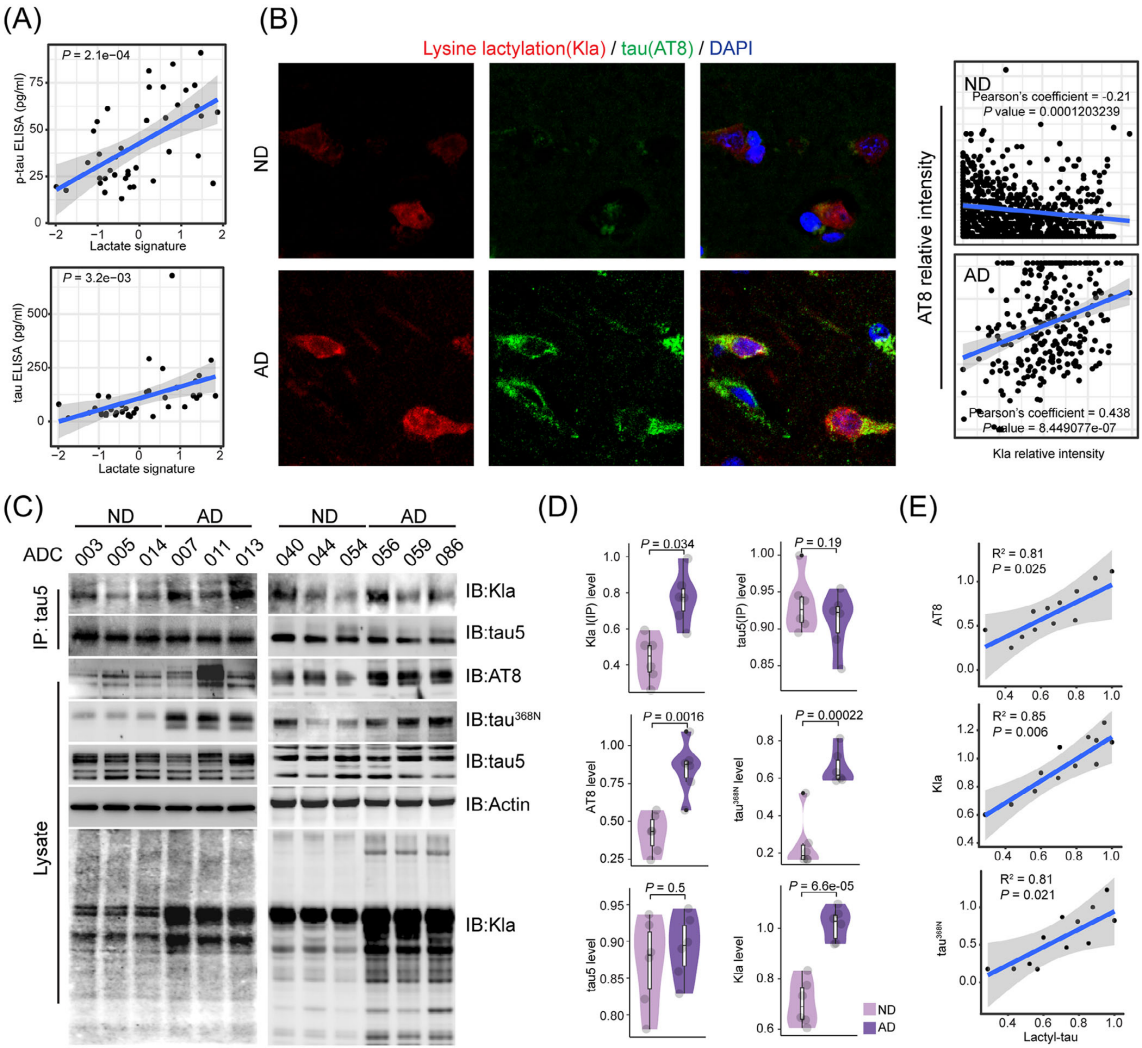
Elevated lactate signature and tau lactylation in AD samples. (A) Association of lactate signature levels with p‐tau (upper) and total tau (lower) levels from CSF proteomics data sets (*n* = 19 control and 20 AD samples). Protein levels were detected by ELISA. (B) Immunofluorescence staining using anti­lactylated lysine (Kla) and anti‐p‐tau (AT8) antibodies on the brain's frontal cortex from post‐mortem human control and AD subjects. Scale bar, 10 µm. Right panel, correlation of AT8 staining intensities with Kla staining intensities in individual cells. (C) Lactylated and total tau levels were determined by immunoprecipitation/immunoblot analysis using anti­lactylated lysine (Kla) and tau5 antibodies, respectively, in six human control and six AD samples. Protein lysates were immunoblotted using p‐tau (AT8), cleaved tau (tau^368N^), tau5, and anti­lactylated lysine (Kla) antibodies. **(D)** Relative levels of the indicated proteins measured by immunoblot analysis in panel C. (**E)** Correlation of the levels of p‐tau (AT8), total tau (tau5), lactylated lysine (Kla), and cleaved tau (Tau^368N^) with lactylated tau in human control and AD samples measured by western blot analysis in C. AD, Alzheimer's disease; CSF, cerebrospinal fluid; ELISA, enzyme‐linked immunosorbent assay; p‐tau, phosphorylated tau.

Next, we investigated a proteomics data set for lactate signature expression in cerebrospinal fluid (CSF) samples from 19 control and 20 AD patients (patient information is provided in Table S3).^16^ Our data show that lactate signature expression is upregulated in AD CSF (Figure S2A,B). Moreover, lactate metabolism–related functions show a profound upregulation as well (Figure S2C). Of interest, our data show that the expression levels of tau and phosphorylated tau detected by ELISA significantly correlate with lactate signature expression (Figure 1A).

Lactate has been shown to induce a PTM in lysine residues on histones and proteins, known as lysine lactylation (Kla)^17^. Immunohistochemistry using an antibody against lactylated lysine (Kla) in histological sections from human patients post‐mortem (Table S1) showed a few cells positive for Kla in ND samples; in contrast, a significant increase in the number of cells positive for Kla was observed in AD samples, especially in neurons (Figure S3A,B).

To examine whether lysine lactylation is associated with tau phosphorylation, we stained sections from human post‐mortem control and AD brains using the Kla antibody with an antibody against p‐tau at Ser202 and Thr205 (AT8). Our data show that proteins had undergone Kla as PTM in cells with p‐tau (Figure 1B).

Because tau often undergoes PTMs, which play crucial roles in regulating its functions,^9^ we tested whether lysine lactylation could occur on tau. Thus, we extracted proteins from postmortem human ND and AD brain samples (Table S1) and isolated tau by immunoprecipitation by using an antibody against total tau (tau5). Our data show that tau isolated from the AD samples exhibited increased levels of lysine lactylation, as detected by the Kla antibody. As expected, tau isolated from the AD samples also shows increased tau cleavage (detected by tau^368N^ antibody) and tau phosphorylation (detected by AT8 antibody) (Figure 1C,D). Moreover, the levels of tau phosphorylation, tau cleavage, and total lactylation in each sample significantly correlate with the levels of tau lactylation in these samples (Figure 1E).

### 3.2 Tau lactylation at K331 is elevated in human AD brain

To identify the lysine lactylation sites in tau associated with AD, we analyzed lysine lactylation as a PTM of tau using a published, publicly available quantitative proteomics (MS) data set by mass spectrometry that analyzed tau PTMs in postmortem control and AD human brain samples from parietal cortex (Brodmann area 39 [BA39]: angular gyrus).^18^ Given that tau PTMs did not exhibit any difference in the soluble fraction,^18^ we focused our analysis on tau lactylation in the insoluble fraction. Due to the corruption of some raw data, which were unusable for analysis, we analyzed data from 47 of 49 AD samples and 38 of 42 control samples.

**FIGURE 2:**
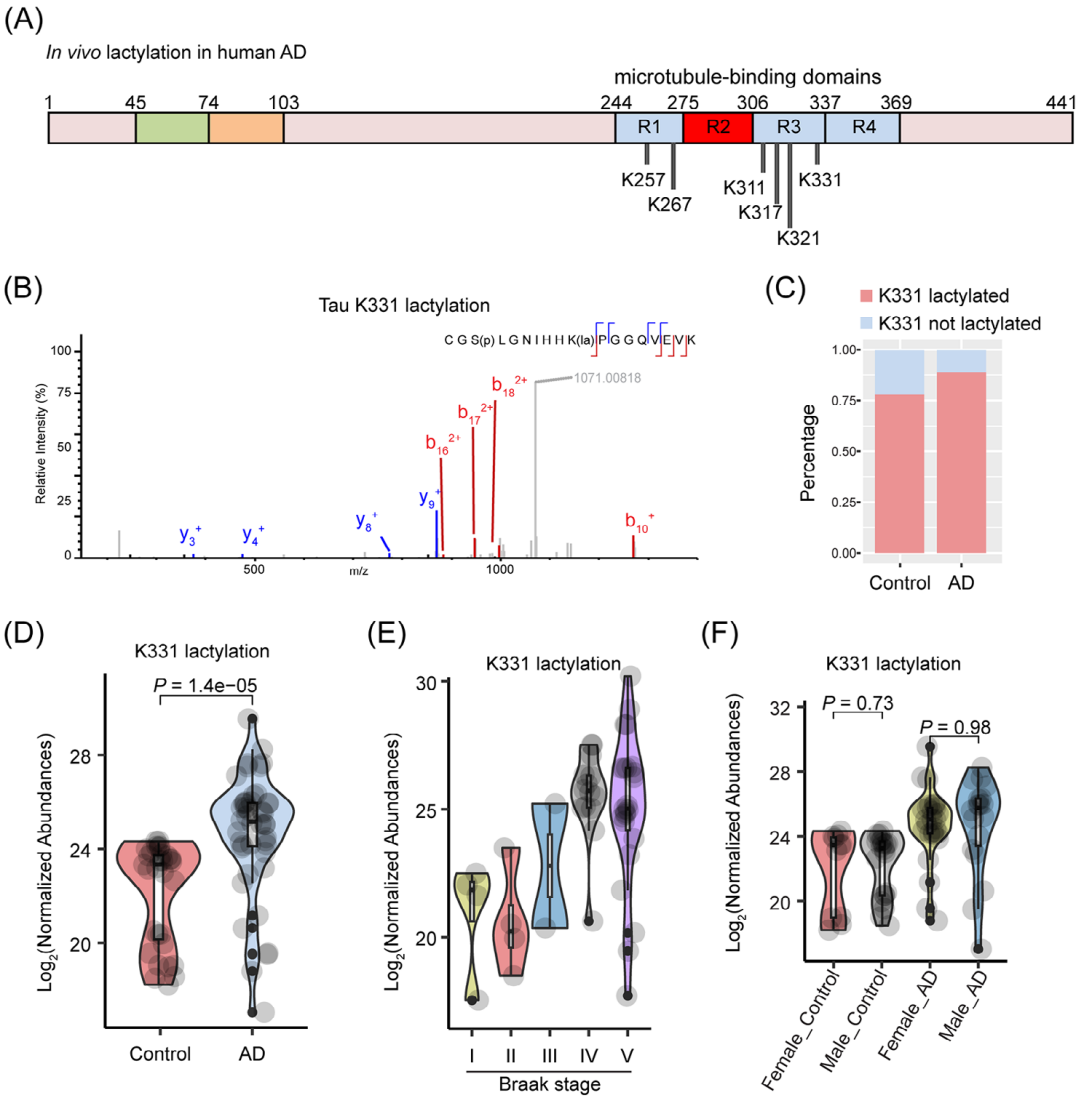
Quantitative proteomics analysis of tau lactylation in human control and AD brain samples. (A) Schematic showing tau‐441 and its key domains. Six lysine sites were identified to be lactylated by MS: K257, K267, K311, K317, K331, and possibly K321. (B) Identification of tau K331 lactylation by MS. (C) Percentages of human control and AD brain samples with or without tau K331 lactylation (*n* = 38 control and 47 AD samples). (D) Log_2_‐normalized abundance of tau K331 lactylation in human control versus AD brain samples (*n* = 31 control samples and 44 AD samples; tau K331 lactylation was not detected in 7 control and 3 AD samples). (E) Log_2_‐normalized abundance of tau K331 lactylation in human control (Braak stages I [*n* = 6], II [*n* = 5], and III [n = 2]) and AD (Braak stages IV [*n* = 14] and V [*n* = 26]) brain samples. (F) Log_2_‐normalized abundance of tau K331 lactylation in female (*n* = 16 control and 28 AD) versus male (*n* = 22 control and 19 AD) brain samples. AD, Alzheimer's disease. MS, mass spectrometry.

Our analysis identified six distinct lactylation sites (lysine residues) on tau, all within the microtubule‐binding domain (Figure 2A). Peptides were identified with combinatorial modifications including lactylation at lysine residue at position 311 (K311) (# peptide spectrum match [PSM] = 3), and K317 or K321 (# PSM = 2) was detected only in the AD samples but not in the control samples. Lactylation at K257 (# PSM = 26) was detected in 41 of 47 (83%) AD samples and 25 of 38 (66%) control samples. Lactylation at K321 (# PSM = 3) was detected in 7 of 47 (15%) AD samples and 3 of 38 (8%) control samples.

Notably, lactylation at K331 (# PSM = 30) was detected in 44 of 47 (94%) AD samples and 31 of 38 (82%) control samples with a significantly increased abundance ratio (log_2_ = 2.3; *p*‐value: 0.00104) (Figure 2B–D). To assess heterogeneity and disease progression reflected by tau lactylation profiles, we observed progressively higher levels of K331 lactylation corresponding to Braak stages (control samples: Braak stages I, II, and III; AD samples: Braak stages IV and V) (Figure 2E). No significant changes were observed between male and female groups (Figure 2F).

Taken together, these data from qualitative and quantitative proteomics by MS establish that tau is lactylated in the human brain and that tau lactylation is elevated in AD (Table S4).

### 3.3 Lactate induces tau lactylation

**FIGURE 3:**
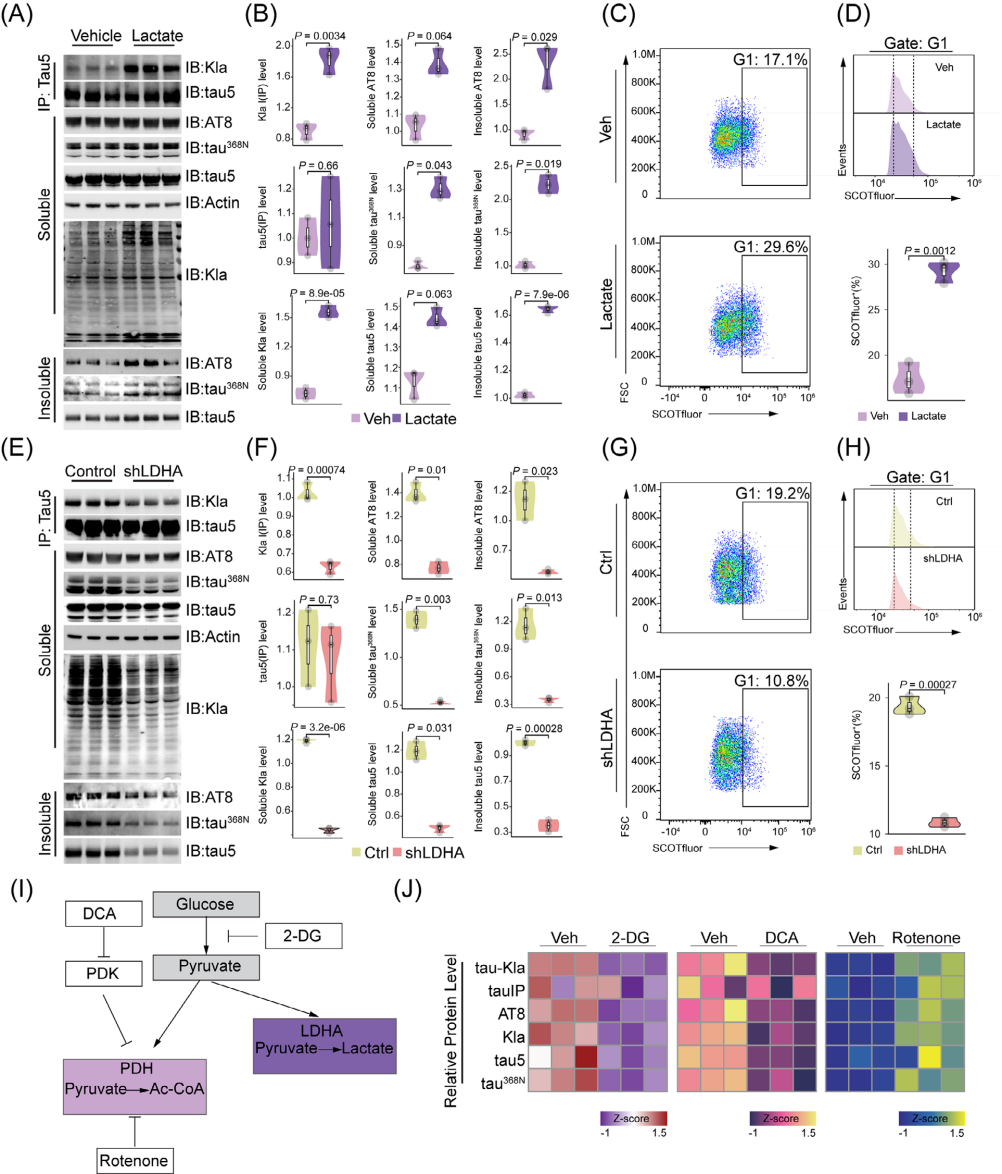
Lactate induces lactylation of tau. (A) Lactylated and total tau levels were determined by immunoprecipitation/immunoblot analysis using anti­lactylated lysine (Kla) and tau5 antibodies, respectively, in HEK 293T cells treated with vehicle or lactate at 2 mM for 24 h. Protein lysates from the soluble and insoluble fractions were immunoblotted using p‐tau (AT8), cleaved tau (tau^368N^), tau5, and anti­lactylated lysine (Kla) antibodies. (B) Relative levels of the indicated proteins measured by immunoblot analysis in A. (C,D) Flow cytometry analysis of lactate levels in tau‐transfected HEK 293T cells treated with vehicle or lactate using lactate probe (SCOTfluor). (E) Lactylated and total tau levels were determined by immunoprecipitation/immunoblot analysis using anti­lactylated lysine (Kla) and tau5 antibodies, respectively, in HEK 293T cells transfected with control and shRNA against *LDHA*. Protein lysates from the soluble and insoluble fractions were immunoblotted using p‐tau (AT8), cleaved tau (tau^368N^), tau5, and anti­lactylated lysine (Kla) antibodies. (F) Relative levels of the indicated proteins measured by immunoblot analysis in E. (G,H) Flow cytometry analysis showed lactate level in HEK 293T cells transfected with control and shRNA against *LDHA* using lactate probe (SCOTfluor). (I) Schematic of the regulation of glycolysis and lactate production by diverse metabolic modulators. (J) Heatmap showing the relative levels of indicated proteins after 2‐DG, DCA, and rotenone treatments measured by immunoblot analysis (Figure S4). The experiment is representative of three independent assays. DCA, dichloroacetate; LDHA, lactate dehydrogenase A.

To examine whether tau lactylation is induced by lactate, we next transfected HEK 293T cells with a plasmid expressing wild‐type tau‐383. HEK 293 cells have been used frequently as a tool cell line for the studies of tau.^19^ A splicing variant human tau with 383 amino acids, tau‐383 includes the four microtubule binding regions (0N4R) but lacks the N‐terminal repeats.^20^ After treating the transfected cells with lactate (2 mM; 24 h), we detected a significantly increased level of lactylation in the immunoprecipitated tau by using the Kla antibody (Figure 3A,B). Of interest,[Fig alz14481-fig-0004] lactate also induced tau phosphorylation, which was detected by using the AT8 antibody, and cleavage, which was detected by the tau^368N^ antibody and occurred most profoundly in the insoluble fraction (Figure 3A,B). We quantified intracellular lactate levels using the SCOTfluor lactate probe, which confirmed that the lactate‐treated cells exhibited increased intracellular lactate accumulation (Figure 3C,D). To confirm these results in neurons, we expressed wild‐type tau in primary mouse neuronal cell culture. After lactate treatment, we detected an increased level of lactylation in the immunoprecipitated tau by using the Kla antibody as well as increased tau phosphorylation in the insoluble fraction (Figure S4). Thus, these data suggest that lactate is an inducer of tau lactylation, phosphorylation, and cleavage.

Lactate dehydrogenase A (LDHA) produces a subunit of the LDH enzyme essential for lactate production in cells.^21^ Because our data detected an endogenous level of tau lactylation in cells (Figure 3A,B), we determined whether this requires LDHA by knocking down *LDHA* using shRNA. After that, we transfected cells with wild‐type tau‐383. Our data show a significantly decreased level of lactylation in the immunoprecipitated tau by using the Kla antibody (Figure 3E,F). These data suggest that inhibiting lactate production reduces tau lactylation. It is notable that the knockdown of *LDHA* also reduced tau phosphorylation and tau cleavage, with these effects being most pronounced in the insoluble fraction (Figure 3E,F). We quantified intracellular lactate levels after *LDHA* knockdown, which confirmed decreased intracellular lactate accumulation (Figure 3G,H). This observation suggests that LDHA‐mediated lactate production induces tau lactylation.

Glucose is a precursor of lactate when glycolysis is not followed by oxidative phosphorylation, leading to the conversion of pyruvate into lactate under anaerobic conditions^22^ (Figure 3I). Given the role of intracellular lactate in driving tau lactylation, we treated HEK 293T cells with the non‐metabolizable glucose analog 2‐DG (Figure 3I), which led to a reduction in tau lactylation (Figure 3M; Figure S4A,B). Concurrently, 2‐DG treatment also downregulated tau phosphorylation and cleavage (Figure 3M; Figure S5A,B). Moreover, because lactate production is regulated by the balance between glycolysis and mitochondrial metabolism,^23^ we investigated whether modulating the activity of key enzymes in these pathways could influence tau lactylation through changes in lactate levels. DCA, an inhibitor of pyruvate dehydrogenase kinase, decreased intracellular lactate^24^ (Figure 3I), leading to reductions in both tau lactylation and tau phosphorylation (Figure 3M, Figure S5C,D). Conversely, treatment with rotenone, a mitochondrial complex I inhibitor that shifts cellular metabolism toward glycolysis,^25^ upregulated tau lactylation (Figure 3M, Figure S5E,F). These findings collectively indicate that endogenous lactate production is a critical determinant of tau lactylation levels.

### 3.4 In vitro lactylation of tau

p300 is an acetyl‐transferase that has been demonstrated to mediate the transfer of lactyl‐CoA to lysine residues in proteins, thereby facilitating lactylation.^26^ Our in silico molecular docking was conducted,^27^ which revealed a strong binding affinity of lactyl‐CoA to p300 (docking energy −20.417 kcal/mol) (Figure S6).

**FIGURE 4 alz14481-fig-0004:**
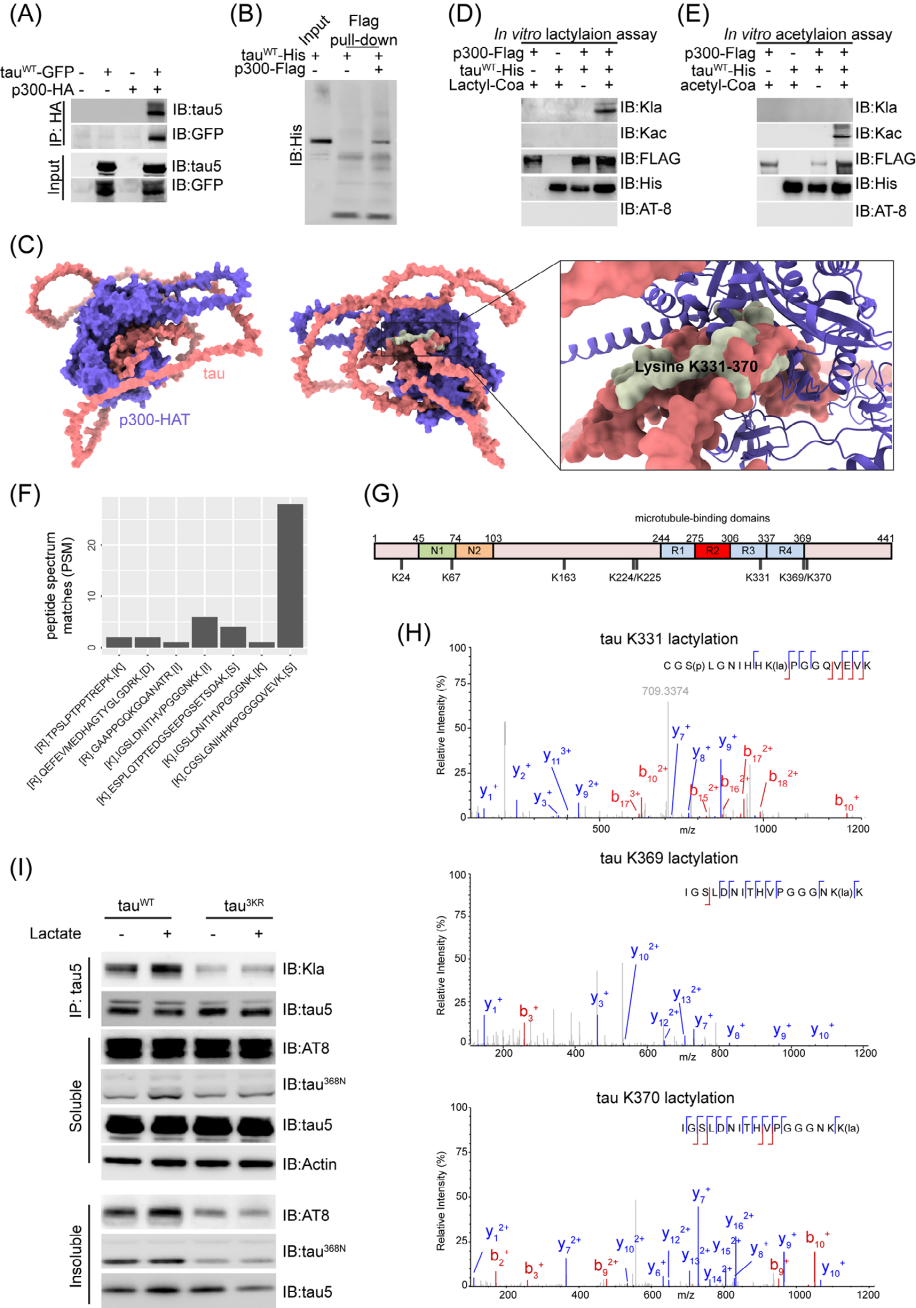
Lactylation of tau by p300 in vitro. (A) HEK 293T cells were transfected with tau (tauWT‐GFP) and p300‐HA. Whole‐cell extracts were collected for immunoprecipitation with the HA antibody, followed by immunoblot analysis by antibodies for total tau (tau5) and GFP. **(B)** Purified tau (tau^WT^‐His) was incubated with Flag‐p300, followed by Flag pull‐down assay and immunoblot with anti‐His tag antibody. (C) The AlphaFold predicted structures of the interaction between p300‐HAT domain and tau. (D) In vitro lactylation assay. Purified tau (tau^WT^‐His) was incubated with recombinant Flag‐p300 with or without lactyl‐CoA, followed by immunoblot analysis using antibodies against anti‐lactylated lysine (Kla), anti‐acetylated lysine (Kac), Flag‐tag (Flag), His‐tag (His), and p‐tau (AT‐8). (E) In vitro acetylation assay. Purified tau (tau^WT^‐His) was incubated with recombinant Flag‐p300 with or without acetyl‐CoA, followed by immunoblot analysis using antibodies against anti‐lactylated lysine (Kla), anti‐acetylated lysine (Kac), Flag‐tag (Flag), His‐tag (His), and p‐tau (AT‐8). (F) PSMs of in vitro lactylated tau in panel D. (G) Schematic showing tau‐441 and its key domains. Eight lysine sites were identified to be lactylated by MS: K24, K67, K163, K224, K225, K331, K369, and possibly K370. (H) Identification of tau K331 lactylation, tau K369, and tau K370 lactylation by MS. (I) Lactylated and total tau levels were determined by immunoprecipitation/immunoblot analysis using anti­lactylated lysine (Kla) and tau5 antibodies, respectively, in HEK 293T cells transfected with wild‐type tau (tauWT) and mutant tau (tau3KR) treated with vehicle or lactate at 2 mM for 24 h. tau3KR harbors K331/369/370R triple mutation. Protein lysates from the soluble and insoluble fractions were immunoblotted using p‐tau (AT8), cleaved tau (tau^368N^), tau5, and actin antibodies. PSMs, peptide spectrum matches.

To experimentally examine whether p300 is a mediator of tau lactylation, we performed co‐immunoprecipitation in HEK 293T cells tranfected with GFP‐tagged tau and HA‐tagged p300. Our data show that tau interacted with p300 (Figure 4A). Pull‐down assay using purified Flag‐tagged p300 and His‐tagged tau further demonstrated a direct interaction between tau and p300 (Figure 4B). Notably, AlphaFold predicted that this interaction specifically involves the HAT domain of p300, which has been shown to facilitate the transfer of the lactyl group from lactyl‐CoA to tau, and the p300–tau interaction particularly targets the K331‐370 residue of tau (Figure 4C).

To further confirm that p300 is a lactyl‐transferase for tau, we incubated purified tau and recombinant p300 proteins with lactyl‐CoA (20 µM), the active form of lactate, in a buffer system optimized for lactylation reactions.^5^ Our results show that tau lactylation occurred exclusively in the presence of tau, p300, and lactyl‐CoA, as detected by the Kla antibody (Figure 4D). Given that p300 is also an acetyl‐transferase, the lysine acetylation (Kac) antibody confirmed the absence of tau acetylation. In addition, the AT8 antibody shows that tau phosphorylation did not occur, consistent with the lack of kinase that phosphorylates tau in the in vitro system. Reciprocally, when acetyl‐CoA was incubated with purified p300 and tau proteins, tau acetylation was induced by the Kac antibody, whereas lactylation was not detected by the Kla antibody (Figure 4E).

To identify the lactylation sites of the in vitro lactylated tau, LC‐MS/MS analysis identified eight lactylated lysine residues in tau, with K331, the most prominent site identified in the human brain upregulated in AD (Figure 2), K369, and potentially K370 showing the highest PSMs (Figure 4F,H, Figure S7, Table S5).

Subsequent mutagenesis studies replacing these lysine residues (K331, K369, and K370) with arginine (tau3KR) profoundly reduced tau lactylation, even under lactate stimulation (Figure 4I). Concurrently, tau3KR shows reduced level of tau phosphorylation and cleavage in the insoluble fraction (Figure 4I). These results confirm that p300 mediates tau lactylation.

### 3.5 Regulation of turnover and cleavage by lactate‐induced tau lactylation

**FIGURE 5:**
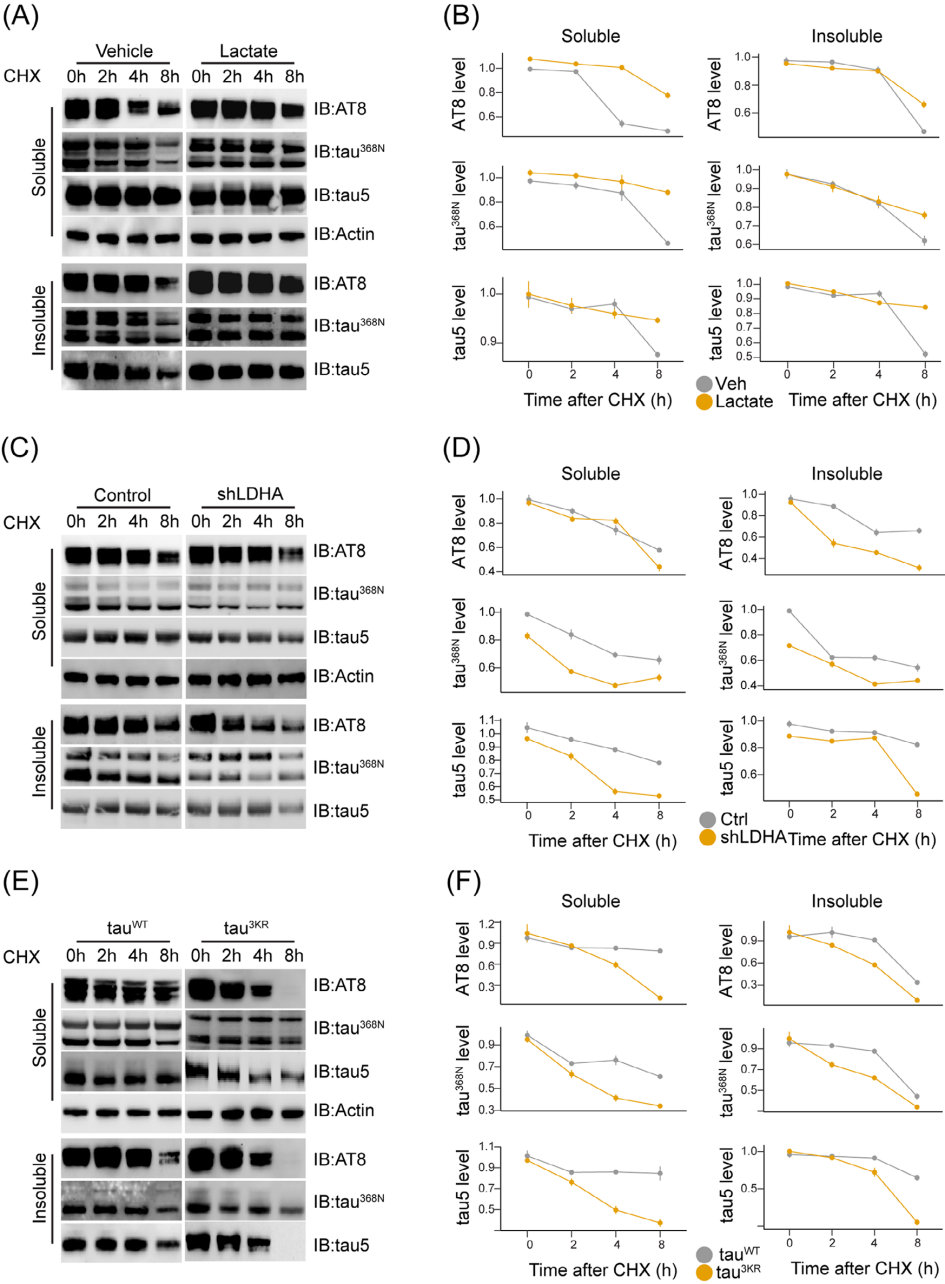
Lactylation regulates tau turnover. (A) HEK 293T cells were transfected with tau^WT^ and treated with cycloheximide (CHX) for indicated times. Phosphorylated and cleaved tau were tested by immunoblot analysis by AT8 and tau^368N^ antibodies, respectively, in the protein lysates from the soluble and insoluble fractions. (B) Relative levels of the indicated proteins measured by immunoblot analysis in A. (C) HEK 293T cells were transfected with control and shRNA against *LDHA* and treated with CHX for the indicated times. Phosphorylated and cleaved tau were tested by immunoblot analysis by AT8 and tau^368N^ antibodies, respectively, in the protein lysates from the soluble and insoluble fractions. (D) Relative levels of the indicated proteins measured by immunoblot analysis in C. (E) HEK 293T cells were transfected with wild‐type tau (tau^WT^) and mutant tau (tau^3KR^) and treated with CHX for the indicated times. Phosphorylated and cleaved tau were tested by immunoblot analysis by AT8 and tau368N antibodies, respectively, in the protein lysates from the soluble and insoluble fractions. (F) Relative levels of the indicated proteins measured by immunoblot analysis in E.

To investigate the function of tau lactylation, we noted that, concurrent with the increase of lactylation of tau, the levels of tau phosphorylation, cleavage, and total tau were also induced by lactate treatment in the insoluble fraction (Figure 2A,B). Thus, we assessed the involvement of lactate‐induced tau lactylation in regulating tau turnover. We compared the turnover rates of wild‐type tau with or without lactate treatment, knockdown of *LDHA*, and tau^3KR^ mutant by the treatment with CHX (Figure 5A–F). Lactate treatment resulted in an increased half‐life of phosphorylated tau, cleaved tau, and total tau (Figure 5A,B). Conversely, knockdown of *LDHA* (Figure 5C,D) or tau^3KR^ mutant (Figure 5E,F) resulted in a decreased half‐life of phosphorylated tau, cleaved tau, and total tau.

**FIGURE 6:**
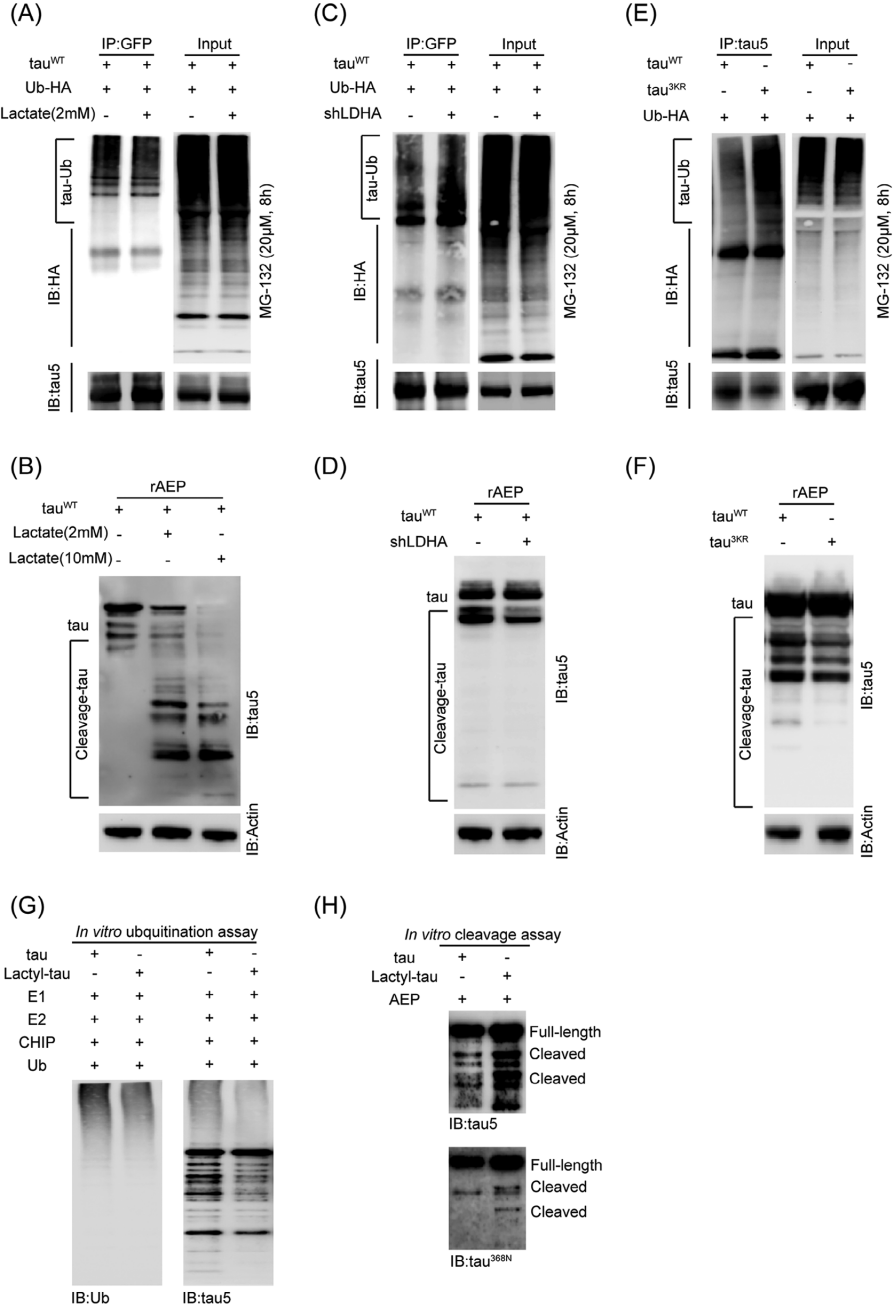
Lactylation regulates tau ubiquitination and cleavage. (A) HEK 293T cells were transfected with tau^WT^‐GFP and HA‐tagged ubiquitin (Ub‐HA). Whole‐cell extracts were collected for immunoprecipitation with anti‐GFP antibody, followed by immunoblot analysis with anti‐HA antibody that detected ubiquitin. (B) HEK 293T cells were transfected with tau^WT^‐GFP and treated with 2 mM and 10 mM lactate for 24 h. Whole‐cell extracts were collected for cleavage assay with rAEP, followed by immunoblot analysis that detected tau by using tau5 antibody. (C) HEK 293T cells were transfected with tau^WT^‐GFP, control, and shRNA against *LDHA*, and Ub‐HA. Whole‐cell extracts were collected for immunoprecipitation with anti‐GFP antibody, followed by immunoblot analysis with anti‐HA antibody that detected ubiquitin. (D) HEK 293T cells were transfected with tau^WT^‐GFP and control and shRNA against *LDHA*. Whole‐cell extracts were collected for cleavage assay with rAEP treatment, followed by immunoblot analysis that detected tau by using tau5 antibody. (E) HEK 293T cells were transfected with tau^WT^‐383, tau^3KR^, and Ub‐HA. Whole‐cell extracts were collected for immunoprecipitation with anti‐tau5 antibody, followed by immunoblot analysis with anti‐HA antibody that detected ubiquitin. (F) HEK 293T cells were transfected with tau^WT^‐383 and tau^3KR^. Whole‐cell extracts were collected for tau cleavage assay with rAEP treatment, followed by immunoblot analysis that detected tau by using tau5 antibody. (G) Purified non‐lactyl‐tau and lactyl‐tau proteins were incubated with ubiquitination proteins (E1, E2, CHIP, and ubiquitin), followed by immunoblotting with anti‐ubiquitin (Ub) and tau5 antibodies. (H) Purified non‐lactyl‐tau and lactyl‐tau proteins were incubated with rAEP, followed by immunoblotting with tau5 and tau^368N^ antibodies.

Ubiquitination of tau marks tau for degradation, typically through the proteasome pathway. To directly test whether lactylation inhibits tau ubiquitination, we transfected HEK 293T cells with expression plasmids encoding GFP‐tagged wild‐type tau‐383 and HA‐tagged ubiquitin. Cells were then treated with lactate to induce lactylation and MG132, a proteasome inhibitor, to block the proteasome‐mediated degradation. After tau was immunoprecipitated by using an anti‐GFP antibody, its ubiquitination was detected by using an anti‐HA antibody. Our data show that lactate inhibited polyubiquitination of tau (Figure 6A). In contrast, knockdown of *LDHA* or tau^3KR^ mutant, which reduced tau lactylation (Figures 3E and 4I), enhanced tau ubiquitination (Figure 6C,E).

In addition, when we incubated cell lysate with recombinant *δ*‐secretase (rAEP), our data show that lactate treatment enhanced AEP‐induced tau cleavage in a lactate dose–dependent manner (Figure 6B). Conversely, knockdown of *LDHA* or tau^3KR^ mutant reduced AEP‐induced tau cleavage in cells (Figure 6D,F).

Finally, we examined whether lactylation of tau directly inhibits its ubiquitination and enhances its cleavage. We incubated control and in vitro lactylated tau (lactyl‐tau) (Figure 4C) with ubiquitin complex proteins in an in vitro ubiquitination assay buffer. Our data show that lactyl‐tau underwent less ubiquitination (Figure 6G). Moreover, by using the in vitro tau cleavage assay, our data show that lactyl‐tau underwent increased cleavage (Figure 6H). Together, these data suggest the roles of lactylation as an important PTM that regulates tau turnover, ubiquitination, and cleavage.

## 4 DISCUSSION

Our findings reveal a novel and significant role of lysine lactylation in tau associated with AD. Elevated lactylation of tau in AD brain samples was detected by proteomics, particularly at the lysine residue K331. The observed upregulation of lactate signature genes and lactylation of tau correlates with increased tau phosphorylation and cleavage, highlighting lactate as a critical mediator of tau pathophysiology. These results suggest that lactylation not only serves as a biomarker for AD but also potentially contributes to tau's pathogenic features, such as its aggregation and impaired clearance. Furthermore, our in vitro experiments indicate that lactate directly induces tau lactylation and affects its turnover, providing new insights into the metabolic regulation of tau and offering potential therapeutic avenues for targeting lactate metabolism in AD.

Our analysis of the published quantitative and qualitative proteomics data set, which includes 47 AD and 38 control human brain samples^18^, revealed that lysine 331 (K331) in tau is a prominent site (Figure 2). Interestingly, our study shows that K331 in tau shows the highest PSM score in our in vitro lactylation assay (Figure 4). K331 is a particularly dynamic site that can undergo multiple types of PTMs, each with distinct implications for tau's function and pathology.^9^ This lysine residue can be modified by ubiquitination, acetylation, methylation, and sumoylation, among others. The ability of K331 to undergo such diverse modifications underscores its critical role in modulating tau's behavior and highlights the complex regulatory mechanisms that govern the involvement of tau in AD and other neurodegenerative diseases involving tauopathies. Understanding the specific conditions under which each modification occurs and their regulatory effects could provide valuable insights into therapeutic strategies aimed at mitigating tau pathology.

In the postmortem human brain samples, certain lactylation sites—K311, K321, and potentially K317—are detected exclusively in the AD but not in control samples. These sites were not identified in p300‐mediated in vitro lactylated tau, which suggests the possibility that lactyltransferases other than p300 may be involved in mediating tau lactylation at these ectopic sites in AD.

In addition, all lactylation sites identified in human samples (Figure 2) are within the microtubule‐binding domains of tau, where positively charged proline‐rich regions interact with the negatively charged microtubule surface. This suggests that tau lactylation might regulate microtubule binding, and in AD, aberrant tau lactylation leads to tau dysfunction.

Although studies have shown that lactate levels increase during early mild cognitive impairment (MCI) but decrease in later stages to control levels, our findings reveal a progressive buildup of lactylated tau at K331 in the brain based on Braak stages (Figure 2). This suggests that the irreversible accumulation of lactylated tau in the insoluble fraction may contribute to disease progression. The accumulation of lactylated tau K331, a residue within tau's microtubule‐binding domain, could interfere with tau's normal role in stabilizing microtubules, leading to impaired neuronal function. Unlike the dynamic fluctuations in lactate levels, the buildup of lactylated tau appears to persist, potentially serving as a pathological marker for the transition from MCI to more severe neurodegenerative stages. This could indicate that lactylation at K331 triggers or exacerbates tau misfolding and aggregation, forming insoluble tau species that further contribute to NFT formation and neuronal toxicity in AD.

In the brain, lactate primarily originates from astrocytic glycolysis, where glucose is either directly converted to lactate to serve as a key energy reserve or transferred to neurons to facilitate metabolism and ATP production. Our study shows that LDHA plays a crucial role in supplying lactate for tau lactylation (Figure 2). Our analysis of the published scRNA‐seq data set revealed that upregulation of *LDHA* gene expression occurred specifically in EX neurons.

Although our results show that tau lactylation is elevated in AD, tau lactylation is also detected in the control brain (Figures 1 and 2), suggesting that this tau PTM plays a physiological role in neuronal function. Indeed, the role of lactate for brain continues to be debated, and what is clear is that lactate is required for physiological function but meanwhile when dysregulated contributes to disease progression. In a recent study, the authors show that restoration of glucose metabolism by inhibition of indoleamine‐2,3‐dioxygenase 1 (IDO1), an enzyme that metabolizes tryptophan to kynurenine (KYN), improves lactate production in AD mouse models and rescues hippocampal memory function.^28^

A primary limitation of our study is the reliance on cell‐based and in vitro experiments to elucidate the effects of tau lactylation. Although these models offer valuable information about tau modification and its association with AD pathology, they cannot fully capture the complexity of tau dynamics in vivo. Future studies using genetic AD mouse models, for example, P301L mice whose hippocampus contains higher levels of lactate at 10 months of age,^29^ will be crucial to explore the physiological and pathological relevance of tau lactylation, and to assess the therapeutic potential of targeting lactate metabolism or tau modifications in vivo.

## CONFLICT OF INTEREST STATEMENT

The authors have no conflicts of interest in the study. Author disclosures are available in the supporting information.

## CONSENT STATEMENT

No human subjects were included in the study. Consent was not necessary for the postmortem human sample study.

## Supporting information



Supporting Information

Supporting Information

Supporting Information

Supporting Information

Supporting Information

Supporting Information

Supporting Information

## Data Availability

The mass spectrometry data have been deposited to MassIVE. The accession number for the data reported in this article is MassIVE MSV000095881. It has also been submitted to ProteomeXchange (PXD055922). Review access can be obtained using the following username and password: Username: MSV000095881_reviewer Password: WangZhang24.
