# Supplementary material for: Lactylation of tau in human Alzheimer's disease brains
Source: Alzheimers Dement. 2024 Dec 30;21(2):e14481. doi: 10.1002/alz.14481 (PMC11851134; doi:10.1002/alz.14481)
Supplement: Supplementary file 1 — Supporting Information [file ALZ-21-e14481-s006.pdf]

Supplementary Information

Lactylation of Tau in Human Alzheimer's Disease Brain

Xiaoyu Zhang[1,2], Yan Liu[1,2], Michaella J. Rekowski[3,4], Ning Wang[1,2,5,6]

[1] Department of Cell Biology and Physiology, University of Kansas Medical Center, 3901 Rainbow Boulevard, Kansas City, KS 66160, USA.

[2] Institute of Reproductive and Developmental Sciences, University of Kansas Medical Center, 3901 Rainbow Boulevard, Kansas City, KS 66160, USA.

[3] Mass Spectrometry/Proteomics Core Laboratory, University of Kansas Medical Center, 3901 Rainbow Boulevard, Kansas City, KS 66160, USA.

[4] Department of Cancer Biology, University of Kansas Medical Center, 3901 Rainbow Boulevard, Kansas City, KS 66160, USA.

[5] Landon Center on Aging, University of Kansas Medical Center, 3901 Rainbow Boulevard, Kansas City, KS 66160, USA.

[6] University of Kansas Alzheimer's Disease Research Center, 4350 Shawnee Mission Parkway, Fairway, KS 66205 USA.

Correspondence

Ning Wang, Department of Cell Biology and Physiology, University of Kansas Medical Center, 3901 Rainbow Boulevard, Kansas City, KS 66160, USA.

E-mail: nwang2@kumc.edu

Xiaoyu Zhang, Department of Cell Biology and Physiology, University of Kansas Medical Center, 3901 Rainbow Boulevard, HLSIC 3094, Kansas City, KS 66160, USA.

E-mail: xzhang8@kumc.edu

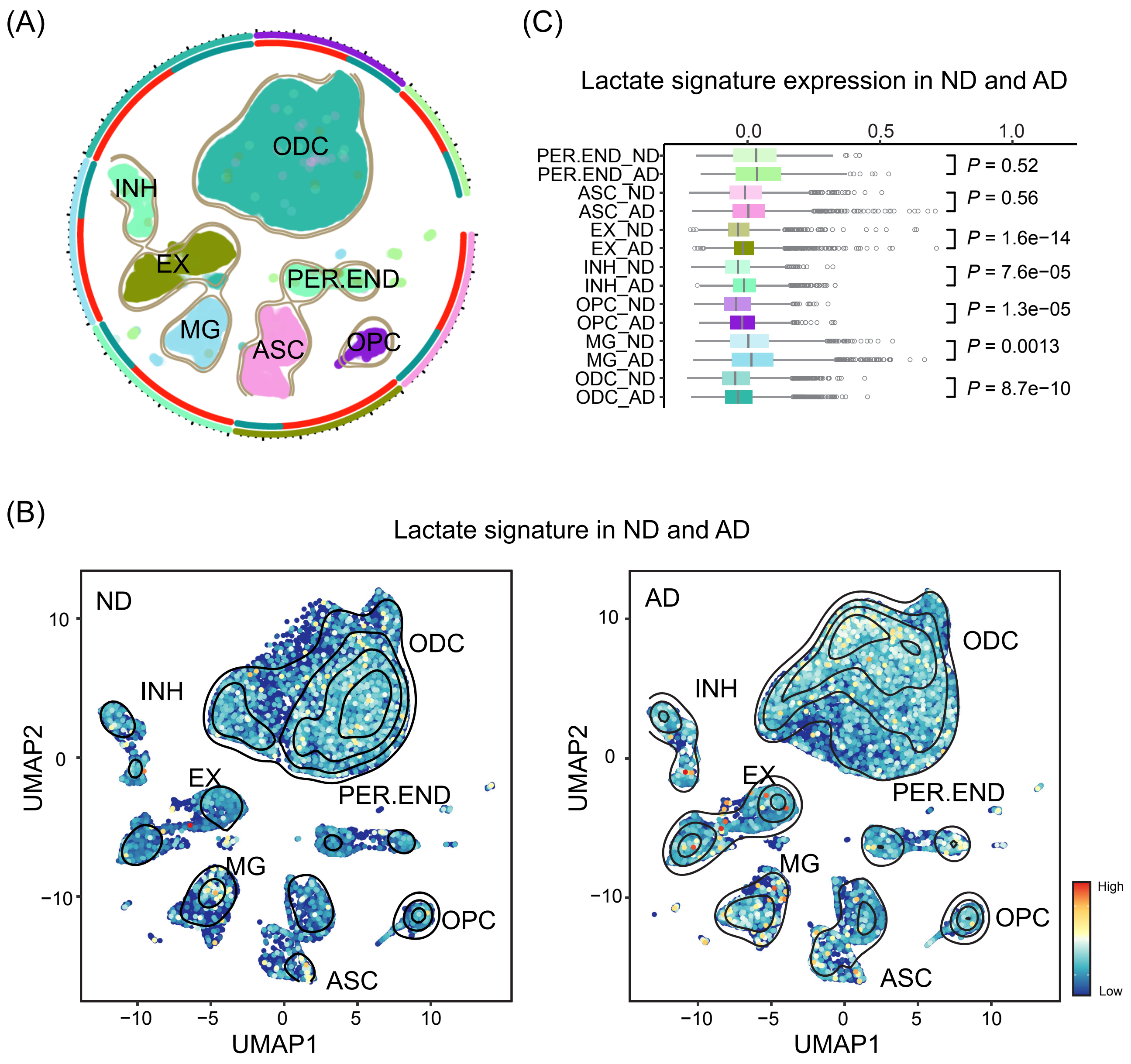

Supplemental Figure 1

Supplemental Figure 1

A, Uniform manifold approximation and projection (UMAP) visualization of integrated projection from human AD and ND brain samples. **B,** Distribution of lactate signature genes in each cluster from human AD or ND brain samples. **C,** Relative level of lactate signature genes in each cluster from human AD or ND brain samples.

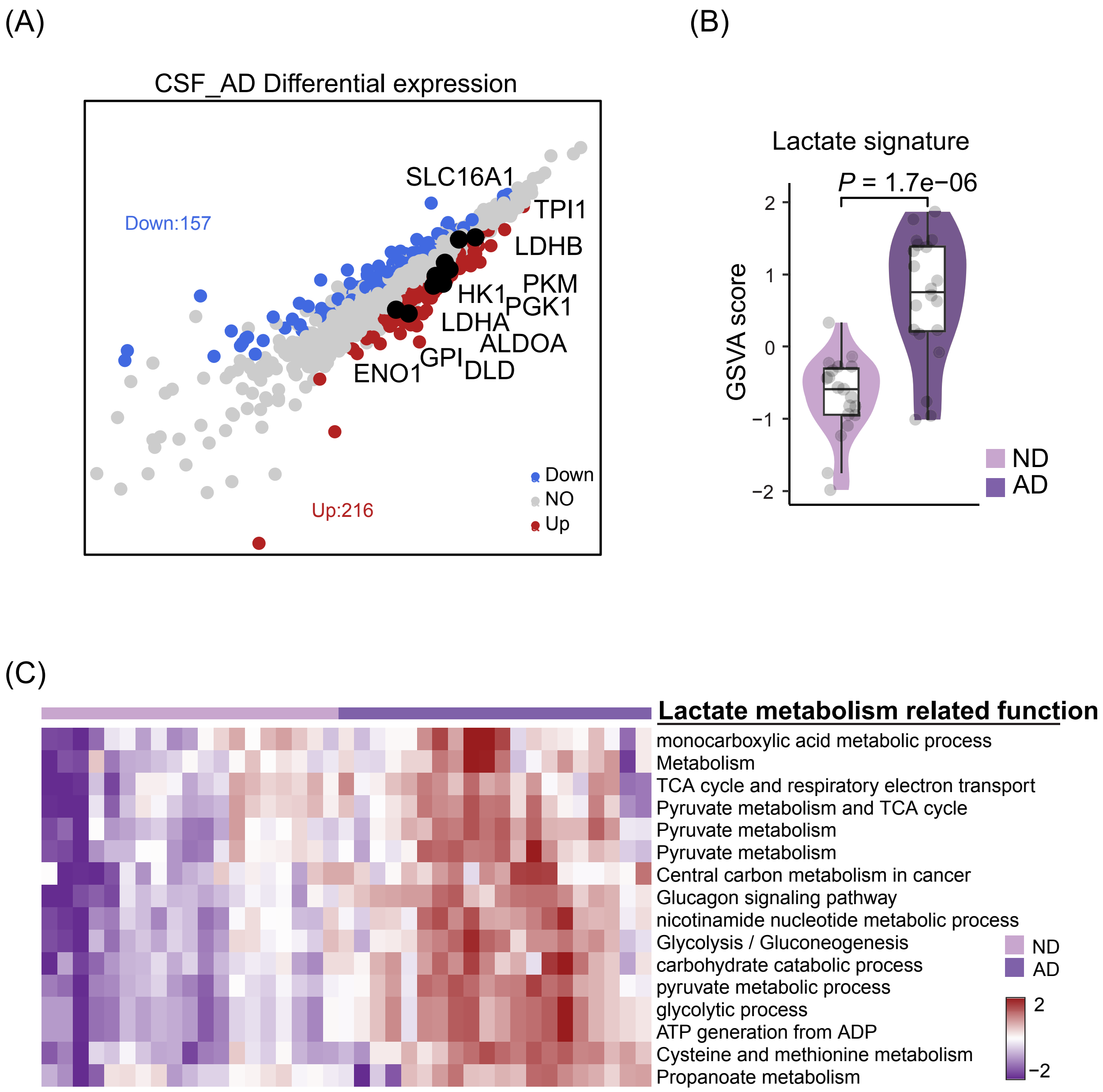

Supplemental Figure 2

Supplemental Figure 2

A, Volcano plot showing lactate signature proteins levels in AD CSF proteomics datasets. **B,** GSVA score of lactate signature proteins levels in AD CSF proteomics datasets. **C,** Heatmap of lactate metabolism-related functions upregulated in AD versus ND samples.

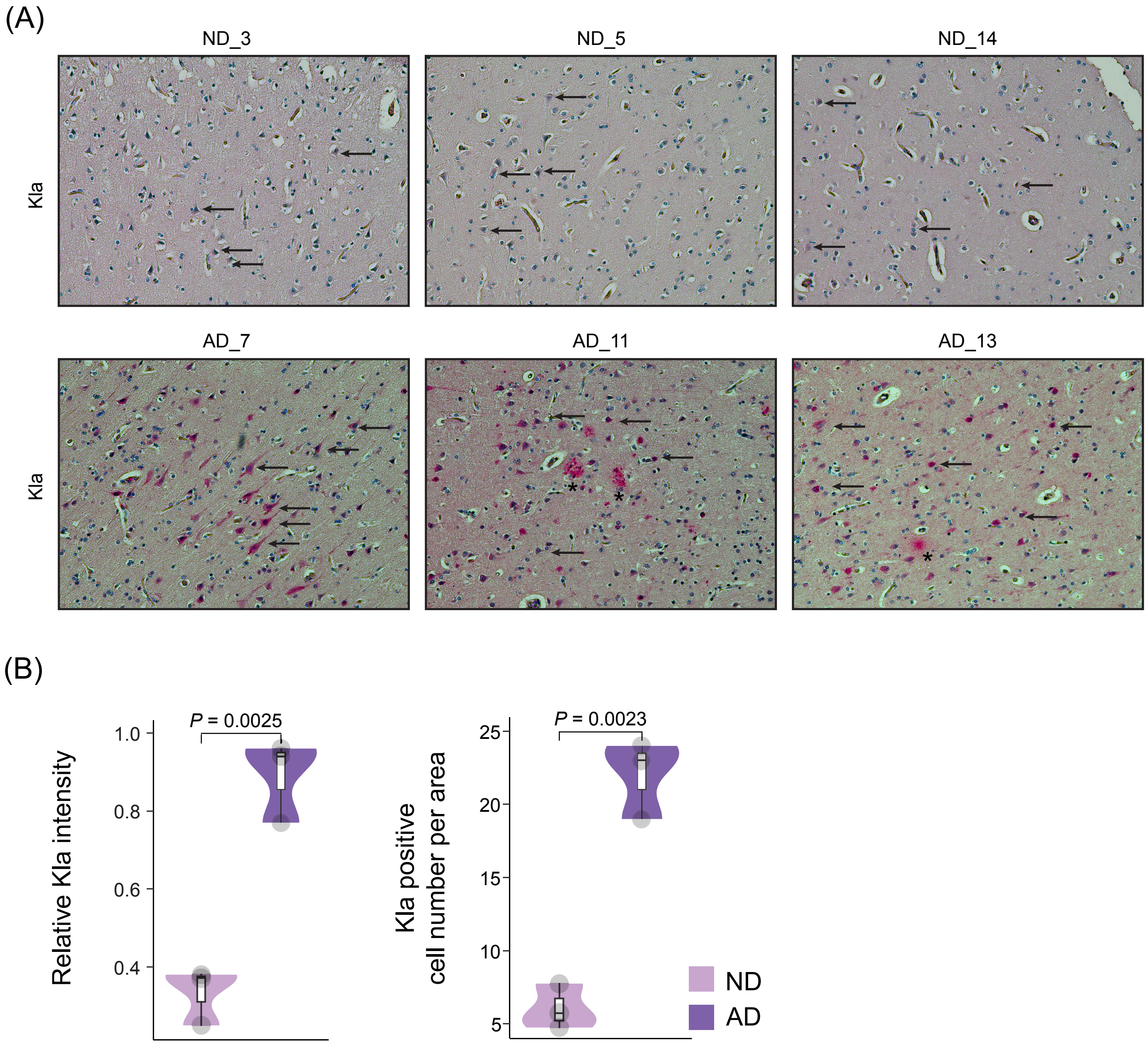

Supplemental Figure 3

Supplemental Figure 3

A-B, Images of immunostaining of pan-Kla in frontal cortex slides from ND and AD individuals. Arrows indicate neurons. Scale bars, 10μm.

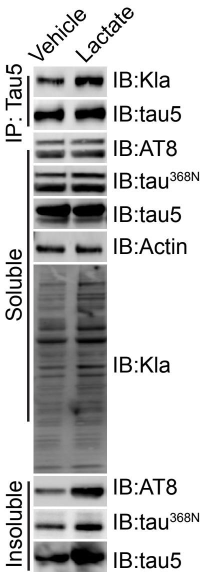

Supplemental Figure 4

Supplemental Figure 4

Lactylated and total tau levels were determined by immunoprecipitation/immunoblot analysis using anti-lactylated lysine (Kla) and tau5 antibodies, respectively, in primary mouse neuronal cell culture treated with vehicle or lactate at 2 mM for 24 hours. Protein lysates from the soluble and insoluble fractions were immunoblotted using phosphorylated tau (AT8), cleaved tau (tau368N), tau5, and anti-lactylated lysine (Kla) antibodies.

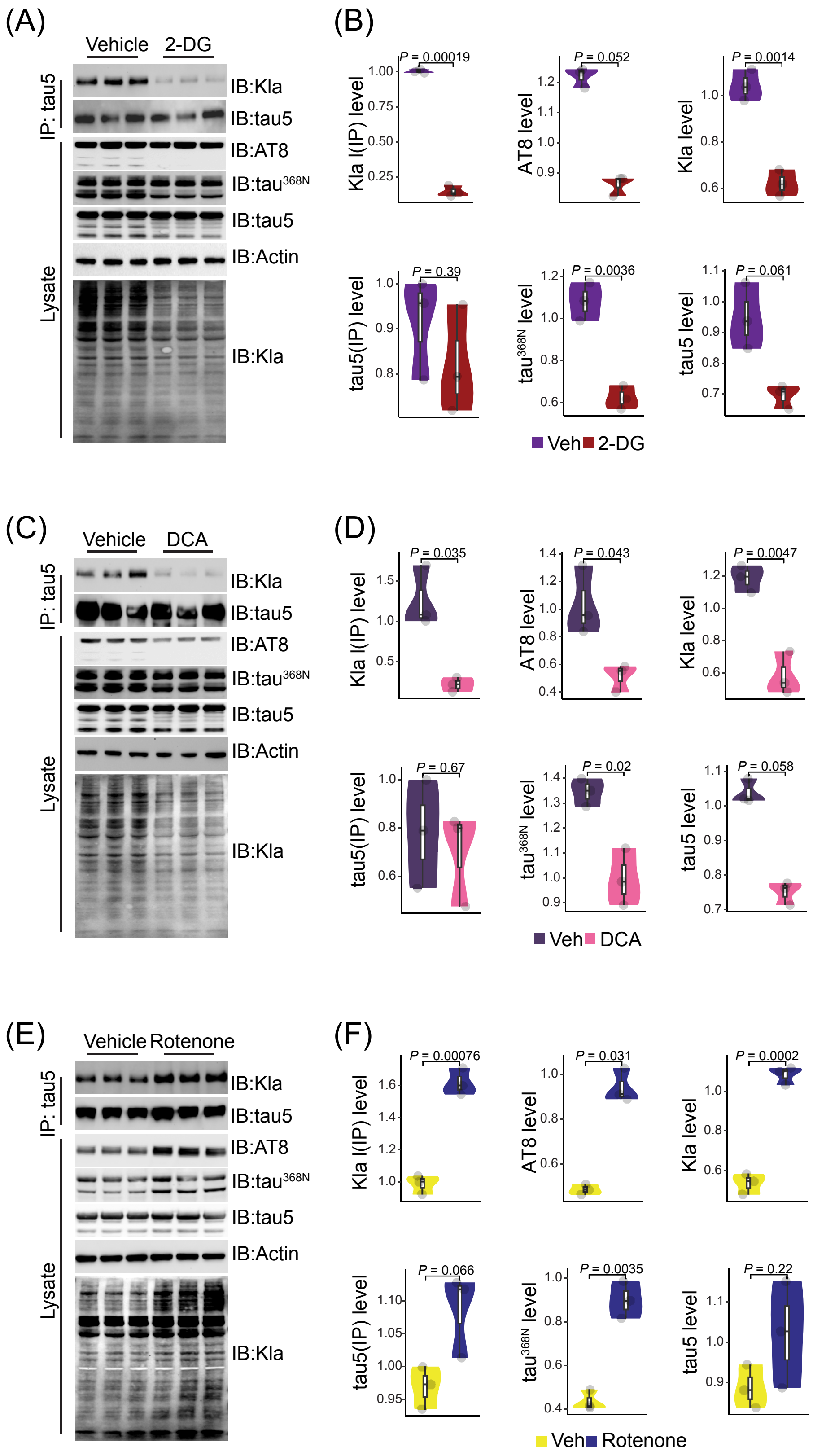

Supplemental Figure 5

Supplemental Figure 5

A-F, Tau associated proteins were analyzed by Western blots of protein samples from HEK 293T cells transfected tau with vehicle, 2-DG, DCA, and rotenone treatment. The experiment is representative of three independent assays.

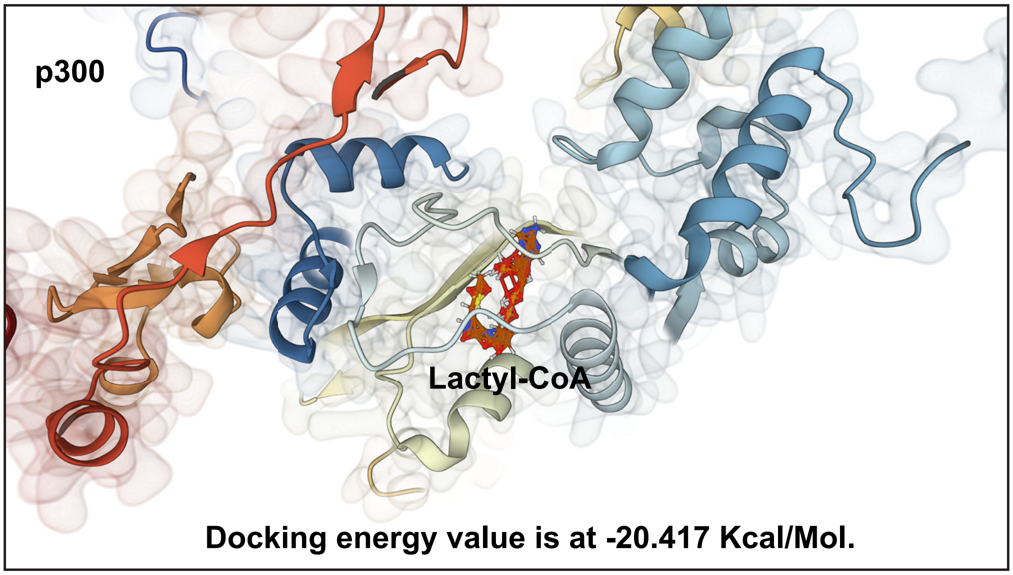

Supplemental Figure 6

Supplemental Figure 6

The cofactor pocket of p300 bound to lactyl-CoA. p300 is shown in cartoon representation. The transfer group in lactyl-CoA is indicated.

MAEPRQEFEVMEDHAGTYGLGDRKDQGGYTMHQDQEGDTDAGLKESPLQT
PTEDGSEEPGSETSDAKSTPTAEDVTAPLVDEGAPGKQAAAQPHTEIPEGTT
AEEAGIGDTPSLEDEAAGHVTQARMVSKSKDGTGSDDKKAKGADGKTKIATP
RGAAPPGQKGQANATRIPAKTPPAPKTPPSSGEPPKSGDRSGYSSPGSPGT
PGSRSRTPSLPTPPTREPKKVAVVRTPPKSPSSAKSRLQTAPVPMPDLKNVK
SKIGSTENLKHQPGGGKVQIINKKLDLSNVQSKCGSKDNIKHVPGGGSVQIVY
KPVDLSKVTSKCGSLGNIHHKPGGGQVEVKSEKLDFKDRVQSKIGSLDNITH
VPGGGNKKIETHKLTFRENAKAKTDHGAEIVYKSPVVSGDTSPRHLSNVSST
GSIDMVDSPQLATLADEVSASLAKQGL

Probable Lactyl-Lys position	tau tryptic peptide sequence
K24	QEFEVMEDHAGTYGLGDRK
K67	ESPLQTPTEDGSEEPGSETSDAK
K87	STPTAEDVTAPLVDEGAPGK
K163	GAAPPGQKGQANATR
K224	TPSLPTPPTREPKK
K225	TPSLPTPPTREPKK
K281	VQIINKKLDLSNVQSK
K331	CGSLGNIHHKPGGGQVEVK
K369	IGSLDNITHVPGGGNKK
K370	IGSLDNITHVPGGGNKK

Supplemental Figure 7

Supplemental Figure 7

Eight sites of tau lactylation were identified in Tau441 protein. K24, K67, K163, K224, K225, K331, K369 and K370 are shown.
