# Supplementary material for: Lactylation of tau in human Alzheimer's disease brains
Source: Alzheimers Dement. 2024 Dec 30;21(2):e14481. doi: 10.1002/alz.14481 (PMC11851134; doi:10.1002/alz.14481)
Supplement: Supplementary file 3 — Supporting Information [file ALZ-21-e14481-s003.docx]

| **Antibodies** | | | Dilution |
| --- | --- | --- | --- |
| Pan-lactyllysine | PTM BIO | PTM-1401RM | 1:1,000 |
| AT8 | Thermo Fisher Scientific | MN1020 | 1:2,000 |
| Tau5 | Sigma | MAB361 | 1:1,000 |
| Tau368N | Sigma | ABN-1703 | 1:5,000 |
| Flag tag | Sigma | F1804 | 1:5,000 |
| HA tag | Sigma | H6908 | 1:5,000 |
| GFP tag | Abcam | ab290 | 1:5,000 |
| Actin | Proteintech | 66009-1-Ig | 1:10,000 |
| His tag | Proteintech | 66005-1-Ig | 1:5,000 |
| Pan-acetylation | Proteintech | 66289-1-Ig | 1:1,000 |
| Goat anti Rabbit | Bio rad | 1706515 | 1:10,000 |
| Goat anti Mouse | Bio rad | 1706516 | 1:10,000 |
| donkey anti-mouse IgG Alexa Flour 488 | Life Technologies | Cat# A21202; RRID:AB_141607 | 1:500 |
| donkey anti-rabbit IgG Alexa Flour 568 | Life Technologies | Cat# A10042; RRID:AB_2534017 | 1:500 |
| donkey anti-goat IgG Alexa Flour 647 | Life Technologies | Cat# A21447; RRID:AB_141844 | 1:500 |

**Supplemental Table 2. Antibodies used in this study.**
